# Inter-reader agreement of quantitative FDG PET/CT biomarkers in lymphoma: a multicentre evaluation of MTV, TLG and Dmax

**DOI:** 10.1186/s12880-025-01937-1

**Published:** 2025-09-17

**Authors:** Elin Trägårdh, Malin Lewold, Jesus Lopez Urdaneta, Måns Larsson, Olof Enqvist, Sally F. Barrington, Mats Jerkeman, Lars Edenbrandt, May Sadik

**Affiliations:** 1 Department of Clinical Physiology and Nuclear Medicine, Skåne University Hospital, Inga Marie Nilssons g 47, Malmö, 20502 Sweden; 2 Department of Translational Medicine, Wallenberg Center for Molecular Medicine, Lund University, Malmö, Sweden; 3 Department of Molecular and Clinical Medicine, Clinical Physiology, Sahlgrenska University Hospital, Sahlgrenska Academy, University of Gothenburg, Gothenburg, Sweden; 4 Eigenvision AB, Lund, Sweden; 5 Department of Electrical Engineering, Chalmers University of Technology, Gothenburg, Sweden; 6 School of Biomedical Engineering and Imaging Sciences, King’s College London and Guy’s and St Thomas’ PET Centre, Kings College, London, UK; 7 Department of Oncololgy, Skåne University Hospital, Lund, Sweden; 8 Department of Clinical Sciences, Lund University, Lund, Sweden

**Keywords:** Lymphoma, Inter-reader variability, FDG PET/CT, Metabolic tumour burden, Total lesion glycolysis

## Abstract

**Background:**

The Deauville score is a key prognostic factor in Hodgkin lymphoma (HL) and diffuse large B-cell lymphoma (DLBCL) during interim and end-of-treatment PET/CT evaluations. However, additional measurements, particularly at baseline, such as metabolic tumour volume (MTV), total lesion glycolysis (TLG), and the maximum distance between hypermetabolic lymphoma lesions (Dmax) may offer enhanced prognostic value. This study evaluates the inter-reader agreement of these metrics to assess their reliability across different physicians.

**Methods:**

This study included 117 patients with untreated HL or DLBCL who had baseline [^18^F]fluorodeoxyglucose PET/CT scans. Nine nuclear medicine physicians independently segmented lymphoma lesions using the online platform Recomia (www.recomia.org), without specific instructions beyond identifying lymphoma-related lesions. MTV, TLG, and Dmax were calculated from these segmentations. MTV was defined as the summed volume in cm^3^, TLG as MTV multiplied by SUVmean and Dmax as the distance between the centroids of the two farthest lesions, measured in the 3D reconstruction. Each patient was segmented by two physicians. Inter-reader agreement was assessed using Spearman correlation coefficients for continuous values and Cohen’s kappa coefficient (κ) for dichotomized values (above/below median).

**Results:**

The mean age of the 117 patients was 50 years (standard deviation 19), 39% female. Median (± interquartile range) values were 321 (± 597) cm^3^ for MTV, 2200 (± 4399) cm^3^ for TLG, and 35 (± 50) cm for Dmax. Spearman correlations between readers were 0.97 for MTV, 0.98 for TLG and 0.72 for Dmax (all *p* < 0.01). Agreement on dichotomized values was 95.7% for MTV (κ = 0.91), 97.4% for TLG (κ = 0.95), 83.8% for Dmax (κ = 0.68).

**Conclusions:**

MTV and TLG demonstrated good inter-reader reliability, even without standardized segmentation protocols. In contrast, Dmax showed moderate variability. These findings support the robustness of MTV and TLG as quantitative biomarkers. For Dmax to be clinically reliable, clearer segmentation guidelines are essential. Especially, inconsistent inclusion of small lesions that may not contribute significantly to MTV, might affect measurement of disease dissemination.

**Supplementary Information:**

The online version contains supplementary material available at 10.1186/s12880-025-01937-1.

## Background

[^18^F]fluorodeoxyglucose ([^18^F]FDG) positron emission tomography/computed tomography (PET/CT) has become an indispensable tool in the management of lymphoma, particularly Hodgkin lymphoma (HL) and diffuse large B-cell lymphoma (DLBCL) [1]. Among its many applications, interim and/or end-of-treatment PET/CT scans are routinely used to assess treatment response, with the Deauville five-point scale serving as a widely accepted assessment method [2]. While the Deauville score provides valuable prognostic information and informs treatment escalation or de-escalation strategies [3–7], it remains a relatively simplistic metric that may not fully reflect the underlying disease biology. Moreover, its use is confined to treatment response assessment, despite evidence suggesting that staging PET/CT also provides prognostic information [8, 9].

In recent years, there has been increasing interest in quantitative PET/CT biomarkers that may provide prognostic information at diagnosis and complement the predictive capabilities of the Deauville score. Parameters such as metabolic tumour volume (MTV), total lesion glycolysis (TLG), and the maximum distance between hypermetabolic lesions (Dmax) have potential to refine risk stratification and predict outcomes in both HL and DLBCL [10–18]. These parameters provide a detailed characterization of tumour burden and spatial dissemination, potentially enhancing clinical decision-making at baseline and during follow-up.

However, the clinical adoption of these quantitative metrics depends on their reproducibility across different readers and institutions. Variability in lesion segmentation, particularly in the absence of standardized protocols, can significantly impact the reliability of MTV, TLG, and Dmax measurements. This issue is especially relevant in multicentre trials and real-world clinical settings, where consistency in image interpretation is essential for accurate prognostication and treatment planning.

Despite growing interest, few studies have systematically evaluated the inter-reader agreement of these quantitative PET/CT metrics in a controlled setting. Evaluating the degree of agreement among nuclear medicine physicians in measuring MTV, TLG, and Dmax is essential for validating their robustness as biomarkers and guiding the development of standardized segmentation protocols.

This study aimed to address this gap by investigating the inter-reader agreement of MTV, TLG, and Dmax in a cohort of patients with newly diagnosed HL or DLBCL. Using manual segmentations performed by multiple experienced nuclear medicine physicians via an online platform, we aimed to quantify the consistency of these measurements.

## Methods

### Patients

This retrospective study included 117 consecutive, newly diagnosed, and untreated patients who underwent staging with [^18^F]FDG PET/CT at Sahlgrenska University Hospital, Gothenburg, Sweden, between 2017 and 2022. Of these, 48 patients with biopsy-confirmed HL, examined between 2017 and 2018, had been previously included in a published study [19]. The remaining 69 patients had biopsy-proven DLBCL and were examined between 2019 and 2022.

The study was approved by the Regional Ethics Committee in Gothenburg (#2019 − 01274), and the need for written informed consent was waived. The study was performed according to the principles of the Declaration of Helsinki and its later amendments.

### PET/CT imaging

Patients fasted for at least 6 h prior to the intravenous administration of [^18^F]FDG. Adults received a dose of 4 MBg/kg (maximum 400 MBq), while pediatric doses were administered according to the EANM Dosage Card (version 5.7.2016). After a 60-minute uptake time, imaging was performed using one of the following integrated PET/CT systems: Siemens Biograph 64 TruePoint, GE Healthcare Discovery MI 5R or GE Healthcare Omni Legend 32. Whole-body PET/CT scans were acquired from the base of the skull to the mid-thigh, with acquisition times of 1–3 min per bed position, depending on the camera system. Images were corrected for scatter and attenuation. A low-dose CT scan (64-slice helical, 120 kV, 30 mAs, 512 × 512 matrix) was obtained and reconstructed with filtered back projection algorithm.

### Image analysis

Quantitative PET metrics (MTV, TLG and Dmax) were derived from manual segmentations. Segmentations were performed by nine nuclear medicine physicians (eight specialists and one resident) from eight different hospitals. Readers were blinded to all clinical data except for the diagnosis of newly diagnosed HL or DLBCL. Lesions were segmented according to published recommendations [20], including:

1. Viable lymph nodes with increased FDG uptake;
2. Focal splenic uptake, regardless of spleen size;
3. Focal uptake in the bone marrow or other extra-nodal sites;
4. Diffuse splenic uptake exceeding liver uptake (spleen/liver ratio > 1.5), in the absence of reactive bone marrow uptake (bone marrow/liver ratio < 1.0).

Segmentations were performed using the cloud-based RECOMIA platform (www.recomia.org), which provided access to CT, PET, fused PET/CT, and maximum intensity projection images [21]. Readers could navigate in sagittal, coronal, and axial planes, adjust PET colour scales and SUV thresholds, and modify CT window settings (e.g. soft tissue, lung, bone). The physicians first visually assessed the scans, then performed segmentations in all slices with suspected lymphoma lesions, using the criteria listed above. No preferred segmentation method was suggested to the physicians. The RECOMIA platform supports fully manual segmentation and segmentation using a manually selected SUV threshold; all segmentations were performed slice by slice. Each patient was independently segmented by two randomly assigned readers, referred to as Reading A and Reading B. A single reader could serve as Reading A for one case and Reading B for another. Six of the readers segmented 12 cases each, and the remaining three readers each segmented 28, 53 and 81 cases, respectively.

MTV (cm³) was defined as the total volume of all voxels labelled as lymphoma. TLG (cm³) was calculated by multiplying the mean standardized uptake value (SUVmean) by the MTV for each lesion and summing across all lesions. Dmax (cm) was defined as the Euclidean distance between the centroids of the two most widely separated lesions in three-dimensional space and was calculated based on the manual segmentations. If one or no lesions were identified, Dmax was set to zero.

### Statistical analysis

Descriptive statistics (mean, standard deviation (SD) and range, or median and inter-quartile range (IQR)) were used to summarize the data. Spearman’s correlation coefficients and 95% confidence intervals (CI) were computed to evaluate the linear relationship between the two manual readings. The mean difference (bias) and 95% limits of agreement (± 1.96 × SD of the differences) between Readings A and B for MTV, TLG, and Dmax were calculated, and Bland-Altman plots were created. To further assess agreement between the readings, the metrics MTV, TLG and Dmax were split by the median and the percentage agreement of values below/above the median were calculated, as well as the Cohen’s kappa coefficient (κ). This method was used to illustrate agreement of a metric being classified as above or below a potential cutoff value for the two Readings. All calculations were also performed when excluding lesions smaller than 3 cm^3^, to evaluate the impact on inclusion/exclusion of small lesions. A p-value < 0.05 was considered statistically significant.

## Results

### Patient characteristics

In the total cohort (*n* = 117), mean age was 50 years (± SD 19, range 7–90), and 39% were female. Among the 48 patients with HL, the mean age was 37 years (± SD 19, range 7–75), with 46% female. In the 69 patients with DLBCL, the mean age was 59 years (± SD 14, range 17–90), and 35% were female.

### Inter-reader agreement for all lesions

The median (± IQR) values for MTV were 336 ± 591 cm^3^ for Reading A and 307 ± 614 cm^3^ for Reading B. For TLG, the corresponding values were 2235 ± 4274 cm^3^ and 2133 ± 4488 cm^3^, respectively. For Dmax, the values were 34 ± 48 cm for Reading A and 35 ± 52 cm for Reading B.

**Fig. 1:**
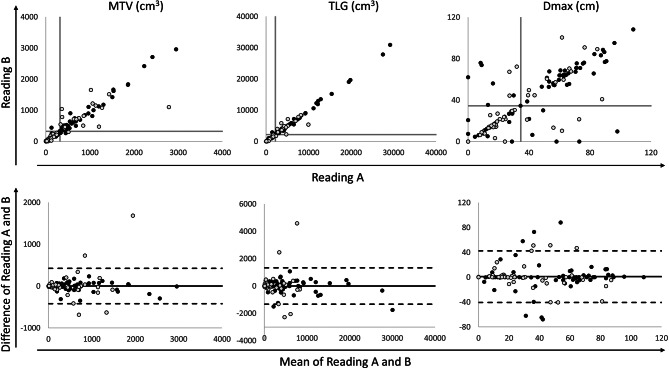
Inter-reader agreement. Scatter (upper row) and Bland-Altman (lower row) plots illustrating inter-reader agreement for MTV, TLG and Dmax between Reading **A** and Reading **B**. White circles represent patients with HL and black circles represent patients with DLBCL. The grey lines in the scatter plots represent the median value for each respective metrics. The solid line in the Bland-Altman plots represents the bias and the dashed lines represent the limits of agreement

Quantitative PET/CT metrics generally showed high consistency between the two independent readings. Spearman correlation coefficients demonstrated strong agreement for MTV (0.97, 95% CI 0.94–0.98) and TLG (0.98, 95% CI 0.97–0.99), but lower for Dmax (0.72, 95% CI 0.55–0.83), (*p* < 0.01 for all metrics). Figure 1 displays scatter and Bland-Altman plots illustrating the variability for MTV, TLG and Dmax. Overall, inter-reading bias was minimal, particularly for TLG, which is expected since the physicians segmenting a case were randomly assigned as Reading A or Reading B. However, the limits of agreement were notably wider for Dmax (Table 1).

**Table 1:** Inter-reading bias and limits of agreement for MTV, TLG and Dmax between reading A and reading B

	Bias	Limits of agreement
MTV (cm^3^)	3.9	± 422
TLG (cm^3^)	-1.6	± 1329
Dmax (cm)	0.1	± 41

Dmax – maximum distance between hypermetabolic tumour lesions, MTV – metabolic tumour volume, TLG – MTV x SUVmean

**Fig. 2:**
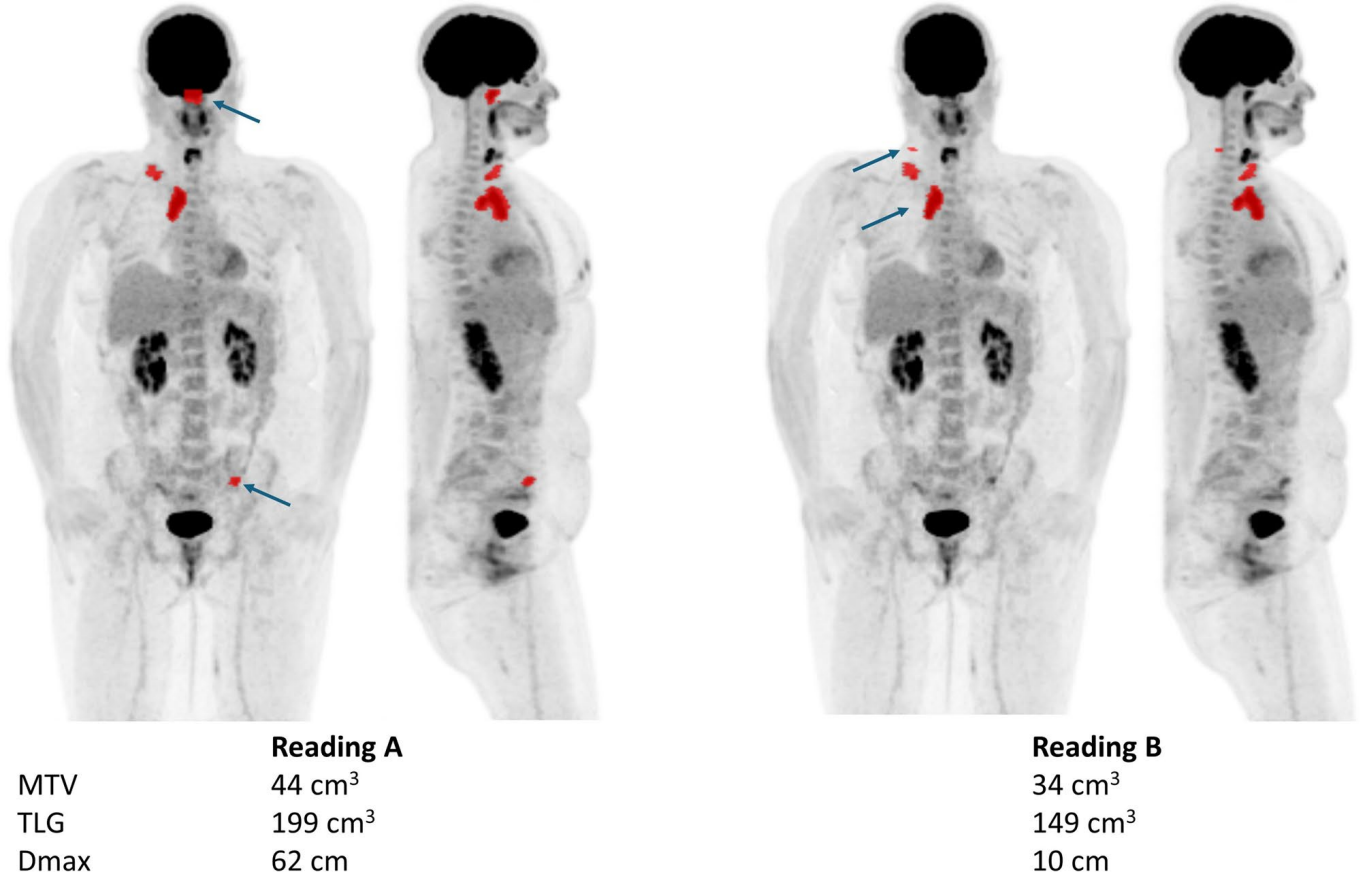
Patient example. Maximum projection intensity PET images (frontal and sagittal views) from one patient with segmentations performed by two physicians (Reading **A** and **B**). The values of MTV, TLG and Dmax are shown below. The arrows point to the two segmentations used for the Dmax calculations

When dichotomizing the metrics based on the median to assess agreement in classifying measurements as above or below the median (the median being used as an example of a cutoff) for the two Readings, the overall agreement was higher for MTV (95.7%, κ = 0.91) and TLG (97.4%, κ = 0.95) than for Dmax (83.8%, κ = 0.68). Table 2 shows the number of patients classified as below or above the median for both Readings (agreement) as well as below the median for one of the Readings and above for the other Reading (non-agreement). The median value was 321 cm^3^ for MTV, 2200 cm^3^ for TLG and 35 cm for Dmax. Figure 2 shows a patient example with segmentations performed by two physicians, with large differences in Dmax and smaller differences in MTV and TLG.

**Table 2:** The number of patients categorized as below/above the median (agreement vs. non-agreement when using the median as cutoff) for MTV, TLG and Dmax for the two readings

**MTV**		Reading B	
		Below median	Above median
Reading A	Below median	56	2
	Above median	3	56
**TLG**		Reading B	
		Below median	Above median
Reading A	Below median	57	1
	Above median	2	57
**Dmax**		Reading B	
		Below median	Above median
Reading A	Below median	49	10
	Above median	9	49

Dmax – maximum distance between hypermetabolic tumour lesions, MTV – metabolic tumour volume, TLG – MTV x SUVmean

### Inter-reader agreement when excluding small lesions

When excluding lesions smaller than 3 cm^3^, the Spearman correlation coefficients for MTV and TLG remained similar to those obtained when all lesions were included (0.96, 95% CI 0.94–0.98 and 0.98, 95% CI 0.97–0.99, respectively), while the coefficient for Dmax increased (0.83, 95% CI 0.71–0.91). All metrics showed statistically significant correlations (*p* < 0.01). A detailed summary of the median (± IQR), bias, and limits of agreement are provided in the Supplementary material.

When dichotomizing the metrics based on the median to assess agreement in classifying measurements as above or below the median for the two Readings, the overall remained unchanged for MTV (95.7%, κ = 0.91) and TLG (97.4%, κ = 0.95) compared to the analysis including all lesions. However, the agreement improved for Dmax (90.6%, κ = 0.81) when small lesions were excluded. Full results are available in the Supplementary material.

## Discussion

This study evaluated the inter-reader agreement of the three quantitative FDG PET/CT biomarkers MTV, TLG, and Dmax in patients with newly diagnosed HL or DLBCL. The findings demonstrate high inter-reader agreement for MTV and TLG. In contrast, Dmax showed greater variability. Excluding small lesions led to improved agreement for Dmax; however, it remained lower than that observed for MTV and TLG. These results suggest that MTV and TLG are more robust and reproducible across readers than Dmax, even in the absence of standardized segmentation protocols. Since it can be anticipated that future use of the metrics in prognostic lymphoma models will be based on cutoff values, we investigated inter-reader agreement by dichotomizing MTV, TLG and Dmax based on the median values.

Quantitative PET/CT biomarkers are increasingly recognized for their potential to enhance risk stratification and guide treatment decisions in lymphoma. While the Deauville score remains the standard for response assessment, it is limited to post-treatment evaluations [2]. In contrast, MTV and TLG offer objective, volumetric assessments of tumour burden that can be applied at baseline and throughout treatment. The high reproducibility of MTV and TLG observed in this study supports their integration into clinical workflows and multicentre trials.

Dmax, which reflects spatial tumour dissemination, has shown prognostic value in previous studies [15–18]. However, its moderate inter-reader agreement in this study highlights the need for standardized segmentation guidelines if this metric is to be used reliably. This is particularly important given that small differences in lesion inclusion, especially for distant or borderline lesions, can disproportionately affect Dmax, without significantly altering MTV or TLG. Also, automated calculations of the centroids and distance between the centroids, as was done in this study, are crucial. If the Dmax had been measured manually, we believe the difference between the readings would be even larger. As can be seen in Fig. 2, as well as in the results when excluding lesions smaller than 3 cm^3^, inclusion or not of small spatially separated lesions have a large impact on Dmax.

Robustness across different imaging systems, reconstruction algorithms, and experience levels is essential for the clinical adoption of advanced PET/CT biomarkers. Various methods exist for delineation of MTV, including fixed SUV thresholds (e.g., SUV ≥ 4.0) and relative thresholds (i.e. 41% of SUVmax in individual lesions). The use of different thresholds leads to different median, and consequently, to different optimal cutoffs to separate patients into high-and low-risk groups. The ideal threshold may vary depending on patient characteristics, lymphoma subtype, and treatment regimen, and should be tailored to specific clinical contexts [20]. In our study, readers were not instructed to use a specific segmentation method, yet MTV and TLG still showed strong agreement. This suggests that even greater consistency could be achieved with standardized or semi-automated approaches.

Manual segmentation is time-consuming, limiting its practicality in clinical workflows. Semi-automated or, preferably, fully automated methods are needed to improve efficiency, as well as to reduce observer dependency and enhance reproducibility across institutions. Artificial intelligence (AI)-based methods can help with this task in the future. We have previously trained an AI-based tool to segment lymphoma lesions and compared the results with manual segmentations [22]. The AI-based tool could be used without major manual adjustments in 69% of HL patients. We are currently working on improving the method.

Previous studies have explored Inter-reader variability in the context of automated or semi-automated workflow. Burggraaff et al. [23] reported improved interobserver reliability for MTV and TLG using a fully automated preselection workflow compared to manual methods. Choi et al. [24] demonstrated high concordance in MTV and TLG measurements between expert readers using different methodologies, further supporting the value of semi-automated tools. Recently an international benchmark has been proposed for MTV measurement using SUV4 and a minimum volume of 3 cm^3^ with high interobserver agreement with a publicly available benchmark dataset against which end-users can test their ability using local software to measure MTV within an acceptable range [25]. To our knowledge, no prior studies have specifically evaluated inter-reader variability for Dmax, making this study a novel contribution in that regard.

A major strength of this study is the inclusion of a relatively large and diverse patient cohort, along with multiple experienced nuclear medicine physicians from different institutions. This enhances the generalizability of the findings. The use of a cloud-based platform (RECOMIA) ensured consistent access to imaging tools and visualization settings, minimizing technical variability.

However, several limitations should be acknowledged. First, the segmentations were performed manually without standardized instructions beyond general recommendations, which may have introduced variability. Second, intra-reader variability was not assessed, which could provide additional insights into measurement consistency. Third, although the readers were instructed to segment all hypermetabolic lymphoma-related findings, the primary purpose of the segmentation was for MTV assessment in separate studies [19, 22]. As a result, small or distant lesions that minimally impact MTV or TLG but significantly affect Dmax may have been inconsistently included. Fourth, since the physicians were randomly assigned cases to segment, the segmentations of one physician may be in Reading A for some cases and in Reading B for some cases. Thus, assessment of “true” inter-reader variability was not possible – for example, the bias between Readings in this study close to 0, but would probably have been larger if one physician was consequently assigned as either Reading A or B. Also, the number of cases segmented by the physicians varied between 12 and 81. In an ideal situation, the cases should have been equally distributed. Fifth, the median value was used as cutoff in this study, but in future prognostic models, other cutoff values are likely to be more relevant.

## Conclusions

This study demonstrates that MTV and TLG are highly reproducible quantitative PET/CT biomarkers in lymphoma, supporting their reliability for use in both clinical practice and research settings. In contrast, the lower inter-reader agreement observed for Dmax highlights the need for standardized segmentation protocols to ensure its consistent application. These findings contribute to the growing body of evidence supporting the integration of quantitative imaging biomarkers into lymphoma management and highlight the importance of harmonization efforts to ensure reproducibility across institutions.

## Supplementary Information

Below is the link to the electronic supplementary material.

Supplementary Material 1

## Data Availability

The data generated or analysed during this study are available from the corresponding author on reasonable request.

## Abbreviations

[^18^F]FDG: [^18^F]fluorodeoxyglucose
AI: Artificial intelligence
DLBCL: Diffuse large B cell lymphoma
Dmax: Maximum tumour dissemination
HL: Hodgkin lymphoma
IQR: Inter-quartile range
MTV: Metabolic tumour volume
PET/CT: Positron emission tomography/computed tomography
SD: Standard deviation
TLG: Total lesion glycolysis
