# Supplementary material for: Inter-reader agreement of quantitative FDG PET/CT biomarkers in lymphoma: a multicentre evaluation of MTV, TLG and Dmax
Source: BMC Med Imaging. 2025 Sep 17;25:368. doi: 10.1186/s12880-025-01937-1 (PMC12442285; doi:10.1186/s12880-025-01937-1)
Supplement: Supplementary file 1 — Supplementary Material 1 [file 12880_2025_1937_MOESM1_ESM.docx]

# Supplementary material

## Results when excluding lesions with a volume < 3 cm^3^

The median (± IQR) values for MTV were 330 ± 575 cm^3^ for Reading A and 297 ± 574 cm^3^ for Reading B. For TLG, the corresponding values were 2136 ± 4207 cm^3^ and 2099 ± 4461 cm^3^, respectively. For Dmax, the values were 21 ± 50 cm for Reading A and 21 ± 54 cm for Reading B.

Supplementary Table 1 shows the bias and limits of agreement. Compared with when all lesions were included, the limits of agreement decreased for all measurements, but the decrease was larger for Dmax.

Supplementary Table 2 shows the number of patients classified as below or above the median for both Readings (agreement) as well as below the median for one of the Readings and above for the other Reading (non-agreement). The median value was 320 cm^3^ for MTV, 2108 cm^3^ for TLG and 21 cm for Dmax.

**Supplementary Table 1.** Inter-reading bias and limits of agreement for MTV, TLG and Dmax between Reading A and Reading B.

|  | **Bias** | **Limits of agreement** |
| --- | --- | --- |
| **MTV (cm^3^)** | 5.3 | ±217 |
| **TLG (cm^3^)** | 3.7 | ±690 |
| **Dmax (cm)** | -0.2 | ±15 |

Dmax – maximum distance between hypermetabolic tumour lesions, MTV – metabolic tumour volume, TLG – MTV x SUVmean

**Supplementary Table 2.** The number of patients categorized as below/above the median (agreement vs non-agreement when using the median as cutoff) for MTV, TLG and Dmax for the two Readings.

| **MTV** |  | Reading B |  |
| --- | --- | --- | --- |
|  |  | Below median | Above median |
| Reading A | Below median | 56 | 2 |
|  | Above median | 3 | 56 |
| **TLG** |  | Reading B |  |
|  |  | Below median | Above median |
| Reading A | Below median | 57 | 1 |
|  | Above median | 2 | 57 |
| **Dmax** |  | Reading B |  |
|  |  | Below median | Above median |
| Reading A | Below median | 53 | 5 |
|  | Above median | 6 | 53 |

Dmax – maximum distance between hypermetabolic tumour lesions, MTV – metabolic tumour volume, TLG – MTV x SUVmean
